# Double Blind Study Investigating the Effect of Different Voice Prostheses on Ease of Swallowing and Residue Post Laryngectomy

**DOI:** 10.1007/s00455-018-9880-0

**Published:** 2018-02-19

**Authors:** Margaret M. Coffey, Neil Tolley, David Howard, Mary Hickson

**Affiliations:** 1 Imperial College Healthcare Trust, SLT Department, Charing Cross Hospital, Ground Floor, South Wing, Fulham Palace Road, London, W6 8RF UK; 2 Imperial College Healthcare Trust, ENT Department, St Mary’s Hospital, Praed Street, London, W2 1NY UK; 3 Imperial College Healthcare Trust, ENT Department, Charing Cross Hospital, Fulham Palace Road, London, W6 8QX UK; 4 Institute of Health and Community, Plymouth University, Drake Circus, Plymouth, Devon PL4 8AA UK

**Keywords:** Laryngectomy, Dysphagia, Voice prosthesis, FEES

## Abstract

Voice prostheses have been examined for their effect on voice production but there is little datum on their effect on swallow function. This study investigated the difference between six commonly available voice prostheses in terms of swallowing. Laryngectomy patients had up to six voice prostheses placed in a random order over two visits. Swallowing was evaluated for each prosthesis using FEES (Fibreoptic Endoscopic Evaluation of Swallowing). After each prosthesis trial, patients self-evaluated their experience of swallowing. Three independent experts indicated which prosthesis they considered best for swallowing for each patient and judged residue on the voice prosthesis and in the upper esophagus. Raters were blinded to participant details, voice prosthesis type and scores of other raters. On patient self-evaluation, scores were equally distributed across all prostheses for swallowing. Experts most frequently chose the Blom Singer Low pressure and Blom Singer Classic Indwelling voice prostheses as best for swallowing but consensus was poor for most patients. Experts found that the Blom Singer Classic Indwelling and the Provox Vega had least residue on the voice prosthesis on thin liquid (*p* ≤ 0.001) and soft (*p* = 0.001), respectively. Experts also found that the Blom Singer Low Pressure had least residue in the upper esophagus on soft consistency (*p* ≤ 0.001). While self-evaluation by patients did not identify a consistently preferred prosthesis for swallow, many patients expressed personal preferences, suggesting benefits to involving patients in the choice of prosthesis. Some voice prostheses may be associated with lower levels of residue on the prosthesis and upper esophagus with certain consistencies.

## Introduction

Laryngectomy surgery involves removal of the larynx in its entirety usually as a treatment for advanced laryngeal cancer. As a result of this surgery, patients lose the ability to communicate in a conventional manner. Surgical voice restoration (SVR) with a voice prosthesis is considered the gold standard for voice rehabilitation after laryngectomy [1, 2]. Since the Blom Singer Duckbill voice prosthesis was introduced [3, 4], there have been numerous improvements to the design and functionality of voice prostheses. These include the introduction of an indwelling prosthesis [5, 6], development of a candida-resistant voice prosthesis [7, 8], enhanced aerodynamic characteristics [9] and changes to insertion methods [10]. As a result, for the patient and clinician, there is currently a wide range of voice prostheses available from which to choose.

The anatomical separation of breathing and swallowing systems post laryngectomy largely eliminates the possibility of aspiration in this patient group. However, laryngectomy patients may experience swallowing difficulty as a result of issues arising from surgery and or radiotherapy ± chemotherapy. These issues include pseudodiverticulum [11–13], stricture [11, 14–16], fistualisation [11, 13, 17], fibrosis [18, 19], impaired pharyngeal propulsion [20]. However, further research is required to identify whether additional factors may affect swallowing after laryngectomy. Anecdotally patients have identified changes in swallowing function when voice prosthesis type is altered. The existing systematic research comparing different voice prostheses has largely focused on perceptual and acoustic measures of voice quality [2, 21–23] or device lifespan [24]. However, to date, the impact of different voice prostheses on swallowing has not previously been investigated.

**Fig. 1:**
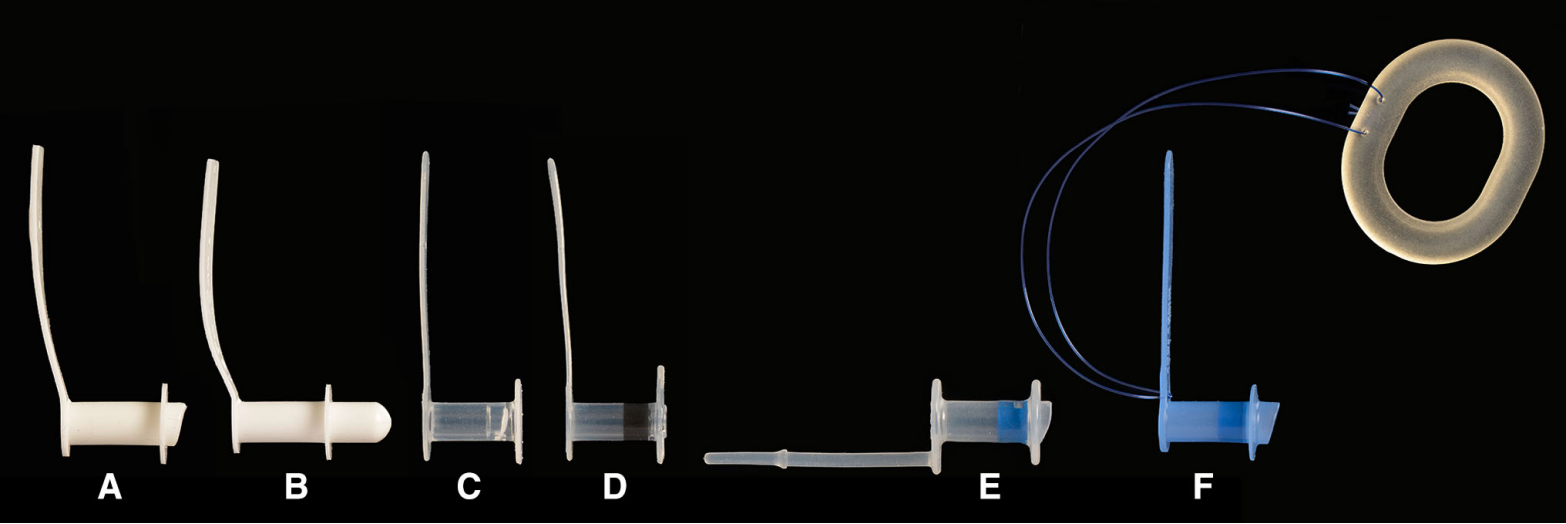
Illustration of voice prostheses used in this study. **a** Blom singer low pressure, **b **Blom singer Duckbill, **c** Blom singer classic indwelling, **d** Blom singer advantage, **e** Provox vega, **f ** Provox NID.

The voice prosthesis is placed in a surgically created puncture between the trachea and esophagus. The distal end of the voice prosthesis sits within the esophagus in the path of bolus flow during swallowing. The configuration of the distal end of each individual prosthesis differs, (Fig. 1) raising the possibility that this part of the prosthesis may interrupt bolus flow or contribute to accumulation of residue during swallowing. While the specific symptoms of swallow impairment post laryngectomy remain poorly understood, there is some evidence that residue is an important symptom which may result in increased effort and time to swallow [13, 20, 25, 26]. In addition, the presence of residue specifically on the voice prosthesis may contribute to leakage and aspiration of swallowed material through the device and into the trachea. Identifying the degree and location of residue post swallow in laryngectomy patients is important as it may influence any future surgical and behavioural interventions to improve swallow.

In this study, FEES was used to evaluate ease of swallowing and residue accumulation with a number of different voice prostheses. FEES was chosen as an evaluation tool as it does not involve radiation exposure and can be safely used to sequentially evaluate swallowing [27].

The aim of this study was to investigate the difference between voice prostheses in terms of ease of swallowing and residue post swallow in 41 post laryngectomy participants. Specific objectives were as follows:

- To investigate whether participants have a preference for a voice prosthesis in terms of ease of swallowing.
- To investigate whether expert raters consider one voice prosthesis as preferable for participants with regard to swallowing function.
- To investigate whether there is a difference between voice prostheses in terms of amount of residue post swallow on the voice prosthesis and in the upper esophagus for all consistencies tested.

## Methods

### Participants

Participants were recruited from the outpatient caseload of head and neck cancer patients at a large NHS tertiary referral centre in the UK, please see participant demographic details, Tables 1, 2. Exclusion criteria included participants without a voice prosthesis, less than 3 months post surgery or post-operative oncological treatment, inability to easily tolerate placement of flexible nasendoscope and documented cognitive dysfunction.

**Table 1:** Patient characteristics

Characteristic	Mean	Min	Max	SD	Median
Age (years)	66.3	43.0	84.7	9.1	64.4
Time since surgery (years)	6.0	4.0	29.0	7.0	8.1

**Table 2:** Surgical and treatment details

Gender	Male 35 (85%) Female 6 (15%)
Ethnicity	Black/Black British (*n* − 5) 12.2% White (*n* − 31) 75.6% Asian or Asian British (*n* − 1) 2.4% Other ethnic groups (*n* − 4) 9.8%
Surgery	Total Laryngectomy (*n* − 30) 73.2% Laryngopharyngectomy with pectoralis major Flap (*n* − 6) 14.6% Laryngopharyngectomy with partial oesophagectomy and jejunum flap (*n* − 3) 7.3% Laryngopharyngectomy with partial oesophagectomy and jejunum and pectoralis major flaps 2 (4.9%)
Myotomy/neurectomy	Yes (*n* − 25) 61% No (*n* − 12) 29% Unknown (*n* − 4) 10%
Closure	Horizontal (*n* − 26) 63.4% Circumferential (*n* − 10) 24.4% Unknown (*n* − 5) 12.2%
Neck dissection	Bilateral (*n* − 10) 24.4% Unilateral (*n* − 8) 19.5% None (*n* − 18) 43.9% Unknown (*n* − 5) 12.2%
Timing of tracheoesophageal puncture	Primary TEP (*n* − 30) 73.2% Secondary TEP (*n* − 11) 26.8%
Radiotherapy history	Pre-operative XRT (*n* − 18) 43.7% Postoperative XRT (*n* − 17) 41.5% Pre and postoperative XRT (*n* − 2) 4.9% None (*n* − 4) 9.8%
Chemotherapy history	Pre op chemo (*n* − 6) 14.6% Post op chemo (*n* − 2) 4.9% No chemo (*n* − 33) 80.5%
Salvage	Yes (*n* − 21) 51.2% No (*n* − 20) 48.8%

### Rating Scale

A scale was developed to enable three expert speech and language pathology raters to judge select findings captured during FEES for each different voice prostheses randomised for use by the participants. Expert raters were chosen because they had at least 5 years’ experience working in large head and neck cancer centres in the UK where they managed laryngectomy patients on a daily basis.

The rating scale consisted of eight continuous visual analog scale questions anchored by the words “minimal” and “severe” to enable experts to rate the degree of residue accumulation on the prosthesis and in the upper esophagus on each consistency. Face validity of the scale was established by surveying a focus group of 6 members of the general public. Content validity of the scale was established through discussion, consultation and agreement with experienced head and neck surgeons and speech and language pathologists.

### Patient Self-Evaluation Questionnaire

Since there was no suitable self-evaluation tool already available, a tool was developed for use in this study. An 11 question Communication and Swallowing with a Voice Prosthesis self-evaluation questionnaire was designed based on feedback from a focus group of 20 laryngectomy patients. This questionnaire had five questions pertaining to swallowing and six questions pertaining to voice. Responses to voice questions were analysed for a separate study. Responses were measured on a five-point Likert scale and the questionnaire contained an additional open question for further comments. The questionnaire was designed to be self-completed by the patient. The face and content validity of the scale was established through discussion and consultation with experienced head and neck surgeons and speech and language therapists.

### Ethics

West London REC granted ethics approval. REC reference number is 10/H0706/25.

### Protocol

Each participant attended two visits. At the start of each visit, data regarding current diet, use of dietary supplements and type of voice prosthesis were recorded, before the current voice prosthesis was removed. The length and diameter of the removed prosthesis were noted and the tracheoesophageal puncture (TEP) was sized. Three prostheses in the appropriate size were then randomly selected from the following:

1. Blom Singer Low Pressure (InHealth Technologies, California, USA) (16 or 20 Fg).
2. Blom Singer Duckbill (InHealth Technologies, California, USA) (16 Fg).
3. Blom Singer Classic Indwelling (InHealth Technologies, California, USA) (16 or 20 Fg).
4. Blom Singer Advantage (InHealth Technologies, California, USA) (16 or 20 Fg).
5. Provox NID (Atos Medical, Horby, Sweden) (17 or 20 Fg).
6. Provox Vega (Atos Medical, Horby, Sweden) (17 or 20 Fg).

**Fig. 2:**
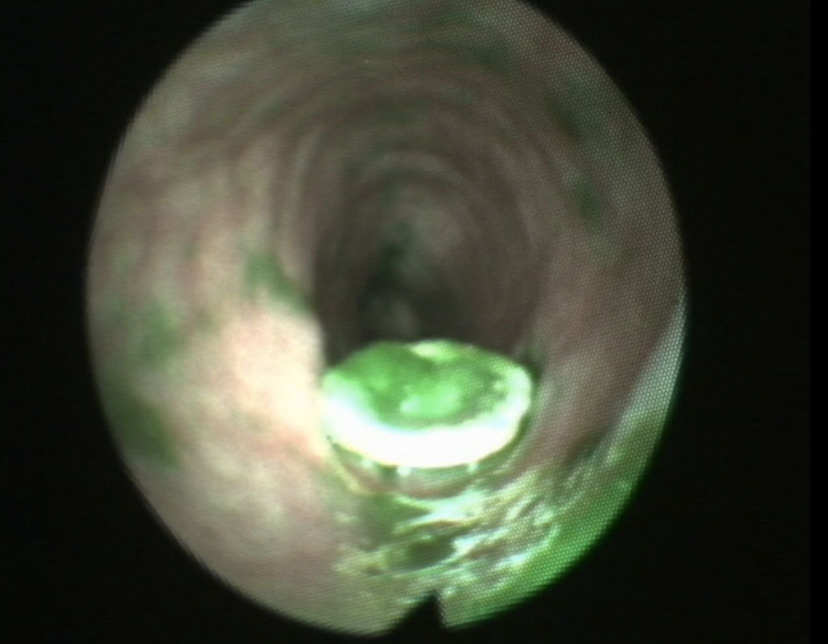
Voice prosthesis and upper esophagus as visualised using nasendoscope

Randomisation was achieved using the “Research Randomizer” programme on the website http://www.randomizer.org/. The initial three prostheses in the randomisation sequence were placed during the first appointment. Participants could see the voice prosthesis but were blinded to the name of the prosthesis and the manufacturer. Each prosthesis was placed according to individual manufacturer’s instructions including use of the gel cap insertion system for Blom Singer prostheses. The absence of central and peripheral leakage for each individual prosthesis was confirmed by asking each participant to take three sips from 200 ml of water coloured with 2 ml of Silver Spoon blue food colouring (British Sugar PLC). The following protocol was used:

A Pentax FNL10RBS flexible nasendoscope (Pentax, Slough, UK) was passed through the right nares where possible. If it was problematic passing the scope through the right nares, the scope was passed through the left nares. Passage of the scope was performed by the primary investigator, a speech language pathologist. The nasendoscope was passed through the pharyngoesophageal segment and advanced to the upper esophagus to enable visualisation of the voice prosthesis. When the voice prosthesis was identified, dynamic recording of the examination using the Kay Pentax Swallow Work Station Model 7127e (Pentax, Slough, UK) was commenced. The nasendoscope remained in place for the duration of each swallow evaluation for each voice prosthesis to generate images for analysis indicating the prosthesis and upper esophagus, see Fig. 2 for a typical example.

Swallow trials for each of the following consistencies were recorded:

1. Thin liquid: 10 ml of 2% semi skimmed milk (Sainsbury’s PLC, London, UK).
2. Puree: 10 ml of Davison’s apple total fruit compote, (Davison’s Canners Limited, Armagh, Northern Ireland) taken from 2 × 90 g pots with 2 ml of Silver Spoon green food dye, (British Sugar PLC, Peterborough UK) added.
3. Soft: 1-cm-thick slice of a medium yellow banana.
4. Solid: ¼ Mc Vitie’s digestive biscuit, (United Biscuits UK Ltd, Middlesex, UK).

Each consistency was given three times for a total of 12 bolus swallows per participant. Water bolus was given after each swallow to rinse any remaining residue. If participants had difficulty swallowing a particular consistency, the number of trials given was reduced to two and occasionally one. At the end of the four consistency trials, the participant was asked to rate their experience of swallow and voice quality using the Communication and Swallowing with a Voice Prosthesis self-evaluation questionnaire. These steps were then repeated with the remaining two prostheses. Once the last prosthesis has been removed, the original prosthesis was replaced.

The second visit commenced as the first and then the remaining two or three prostheses in the randomisation sequence were placed. This depended on the French gauge of the patient’s resident prosthesis; the Blom Singer Duckbill voice prosthesis is not available in 20 Fg so could not be used if the patient required this size. The consistency trials and self-evaluation were then repeated as before.

### Methods of Image Data Collection

The dynamic recordings of each individual swallow were extracted in AVI format. Participant number and consistency were then added as labels to these files and identifying information was removed using Apple Final Cut Pro version 6.06, (Apple Inc., California, USA) which is a non-linear editing software application. Endoscopy exams were re-exported in high definition 720pHD format to be viewed using Apple QuickTime player. The individual exams for each participant were then placed in a random order on three Western Digital My Passport 500 GB super speed USB external hard drives (Western Digital, California, USA) before presentation to expert raters. Raters were blinded to both participant details and prosthesis. From these data, raters gave an independent evaluation of each FEES exam based on all swallows for each consistency. Following evaluation of FEES exams for each prosthesis, participant and consistency, raters were asked to indicate the prosthesis they considered “best” for swallow for that participant.

### Analysis

Data were entered and analysed in IBM SPSS (Statistical Product and Service Solutions), version 23, IBM Armouk, New York.

### Participants’ Preference for a Voice Prosthesis for Swallow

Five questions relating to swallow from the Communication and Swallowing with a Voice Prosthesis self-evaluation questionnaire were scored. Each question was assigned a score from 1 to 5 with a higher score indicating a more negative evaluation. These scores provided a total swallow score for each prosthesis. The possible highest total score for each prosthesis was 25. These data represented a single factor, repeated measures design with six experimental conditions. Therefore, these data required analysis using the non-parametric measure Friedman Two-Way Analysis of Variance by Ranks. Median descriptive scores were elicited for each prosthesis.

### Expert Raters’ Preference for a Voice Prosthesis for Swallow

A consensus score for best voice prosthesis for swallow for each participant was calculated from the ratings of the three clinicians; a consensus was that two or more raters considered that prosthesis best for swallow for that particular patient.

### Rating Scale Reliability

Intra- and inter-rater reliability for the rating scale was examined for all three raters using Intraclass correlation coefficients (ICC). A 2-way mixed model was chosen [28]. As 0.6 ICC has previously been indicated as signifying a useful [29] and good [30] level of agreement, this interpretation was used to bench mark reliability data.

### Residue on the Voice Prosthesis and Upper Esophagus

Each participant had up to 6 voice prostheses placed. As all physiological parameters other than type of voice prosthesis were equal for each participant, three expert raters judged residue, which was directly attributable to the presence of the prosthesis. A consensus score for amount of residue observed on each voice prosthesis and in the upper esophagus, on each swallow consistency, for each participant was calculated from the ratings of the three raters; agreement between two or all was considered a consensus. This was calculated by measuring agreement for whether or not residue is present. If residue was present, agreement for amount of residue was measured.

Each continuous consensus value was then checked for normal distribution using P–P plots. Once normal distribution was confirmed, repeated measures of analysis of variance was chosen as the method of analysis. When significant, post hoc analysis using Bonferroni correction was undertaken to see exactly where differences lay in the data. Pairwise comparisons were undertaken as each participant used each prosthesis.

## Results

Forty-two laryngectomy participants were screened and consented for this study. One participant was excluded because he failed to attend the second appointment. Each of the participants agreed to attend two appointments within a 7-day period and trial up to six voice prostheses. Forty participants attended two appointments within a 48-h period with one participant attending two appointments within a 72-h period. All participants tolerated passage of nasendoscope easily and without complications. Thirty-two participants habitually used a 16 Fg prosthesis and had up to six 16 Fg or 17 Fg prostheses placed. Nine participants habitually used a 20 Fg prosthesis and had up to five 20 Fg prostheses placed. Most participants trialled all prostheses except for six participants. Five of which each declined a trial of one prosthesis, while one participant declined two prostheses. In all cases, prostheses were declined due to patient fatigue. The total number of prostheses analysed was 230.

All participants used Blom Singer voice prostheses prior to recruitment to this study. Ninety-seven point six percent (*n* = 40) were primarily tracheoesophageal speakers, 2.4% (*n* = 1) chose to use primarily esophageal speech although this participant had good functional tracheoesophageal voice. Further details regarding participants in this study are outlined in Tables 1 and 2.

### Participants’ Preference for a Voice Prosthesis for Swallow

Median descriptive scores were elicited from the Communication and Swallowing with a Voice Prosthesis self-evaluation questionnaire, for each prosthesis, as shown in Table 3, showing no significant difference in the ratings between the prostheses overall.

**Table 3:** Median descriptive scores for each prosthesis and Friedman test results—Subject preference for voice prosthesis for swallow

Prosthesis type	*N*	Percentiles—scores for swallow		
		25th	50th (median)	75th
Blom Singer Duckbill	32	12	14	17
Blom Singer low pressure	41	10	13	15
Blom Singer Classic Indwelling	41	10	14	15
Blom Singer Advantage	41	12	12	14
Provox Non-Indwelling (NID)	40	10	13	16
Provox Vega	35	10	13	14
Friedman test		Chi Square 7.89		
		df 5		
		Significance 0.16		

Five questions relating to swallow from a “Communication and Swallowing with voice prostheses self-evaluation questionnaire” were scored. Scores from each swallow question were then added to provide a total swallow score for each prosthesis for each individual subject. The higher the score achieved, the more negatively subjects evaluated swallow. Maximum possible score = 25

### Expert Raters Preference for a Voice Prosthesis for Swallow

The results are shown in Table 4, where a frequency of 4 means that particular prosthesis was agreed to be the “best” for four patients.

**Table 4:** Frequency analysis of expert raters consensus of best prosthesis for swallow

Prosthesis	*n*	Frequency	Percentage of sample
Blom Singer Duckbill	32	0	0
Blom Singer Low pressure	41	4	9.7
Blom Singer Classic Indwelling	41	4	9.7
Blom Singer Advantage	41	0	0
Provox NID	40	3	7.5
Provox Vega	35	2	5.7
No consensus best prosthesis for swallow	41	28	68.3

This analysis indicates that the Blom Singer low pressure and Blom Singer classic indwelling were most frequently chosen (9.7% of sample) as best prosthesis for swallow. Neither the Blom Singer duckbill nor the Blom Singer Advantage was chosen on any occasion by clinicians as best for swallow. But for most patients (68.3%) no consensus was reached among raters.

### Rating Scale Reliability

Intra-rater reliability was > 0.6 (Intraclass Correlation Coefficient (ICC) for 5/8 (62%) questions. Inter-rater reliability > 0.6 ICC for 3/8 questions (37%).

### Residue on the Voice Prosthesis and Esophagus

These data are shown in Table 5 for residue on the voice prosthesis and Table 6 for residue in the upper esophagus.

**Table 5:** Repeated measures of analysis of variance—voice prosthesis residue expert rating

Prosthesis type	Consistency	Mean^a^	SE	Df	95% confidence interval	
					Lower bound	Upper bound
Blom Singer Duckbill	Thin liquid	40.45	4.24	33.95	31.84	49.07
	Puree	48.83	3.42	30.63	41.85	55.8
	Soft	57.02	4.56	35.36	47.76	66.27
	Solid	50.93	3.90	33.99	43.01	58.85
Blom Singer Low Pressure	Thin liquid	31.19	3.76	35.30	23.55	38.83
	Puree	48.56	4.39	37.40	39.67	57.45
	Soft	49.89	4.66	35.65	40.43	59.34
	Solid	50.35	4.47	33.65	41.26	59.44
Blom Singer Classic Indwelling	Thin liquid	31.19	3.76	35.30	23.55	38.83
	Puree	50.20	4.72	39.77	40.65	59.75
	Soft	46.76	4.73	34.80	37.16	56.37
	Solid	43.30	4.46	39.16	34.29	52.32
Blom Singer Advantage	Thin liquid	44.94	3.30	35.69	38.24	51.65
	Puree	58.82	3.77	40.44	51.21	66.44
	Soft	49.17	4.21	39.85	40.66	57.69
	Solid	50.97	3.99	40.19	42.91	59.04
Provox Non-Indwelling	Thin liquid	39.37	3.78	31.25	31.67	47.08
	Puree	55.76	4.22	38.09	47.21	64.32
	Soft	58.09	4.39	37.76	49.19	66.98
	Solid	50.68	4.39	39.46	41.8	59.57
Provox Vega	Thin liquid	41.67	3.73	23.56	33.96	49.37
	Puree	49.86	4.34	34.53	41.05	58.67
	Soft	43.32	4.67	25.49	33.71	52.94
	Solid	47.38	4.67	33.6	37.89	56.87

^a^From 0 to 100 mm scale where 0 = minimal residue and 100 = severe residue

**Table 6:** Repeated measures of analysis of variance—upper esophageal residue expert rating

Prosthesis type	Consistency	Mean^a^	SE	Df	95% confidence interval	
					Lower bound	Upper bound
Blom Singer Duckbill	Thin liquid	44.47	3.61	30.88	37.12	51.83
	Puree	53.3	2.79	35.56	47.64	58.96
	Soft	60.11	3.80	32.02	52.37	67.85
	Solid	48.8	3.47	33.89	41.74	55.86
Blom Singer Low Pressure	Thin liquid	43.88	3.13	40.88	37.57	50.2
	Puree	53.89	3.94	41.02	45.94	61.85
	Soft	46.61	3.64	39.66	39.26	53.97
	Solid	44.6	3.49	39.68	37.53	51.67
Blom Singer Classic Indwelling	Thin liquid	40.27	2.91	41.03	34.39	46.15
	Puree	58.7	3.92	40.71	50.79	66.62
	Soft	50.30	3.72	38.42	42.78	57.82
	Solid	43.30	4.46	39.16	34.29	52.32
Blom Singer Advantage	Thin liquid	44.14	3.06	41.00	37.96	50.32
	Puree	56.73	2.91	41.00	50.85	62.62
	Soft	50.76	3.95	38.76	42.76	58.76
	Solid	50.97	3.99	40.19	42.91	59.04
Provox Non-Indwelling	Thin liquid	42.64	3.41	41.43	35.76	49.53
	Puree	51.41	3.19	40.88	44.95	57.86
	Soft	54.22	3.88	37.62	46.36	62.07
	Solid	50.68	4.39	39.46	41.8	59.57
Provox Vega	Thin liquid	44.15	3.22	31.68	37.59	50.72
	Puree	51.06	4.03	36.05	42.88	59.23
	Soft	40.8	3.63	33.98	33.42	48.17
	Solid	47.38	4.67	33.6	37.89	56.87

^a^From 0 to 100 mm scale where 0 = minimal residue and 100 = severe residue

Type III tests of fixed effects indicated a significant difference between voice prostheses in terms of post swallow residue on the voice prosthesis on thin liquids (*p* ≤ 0.001) and soft consistencies (*p* = 0.001). For thin liquids, the Blom Singer Advantage had most residue on the prosthesis with least residue on the Blom Singer Indwelling. On soft, the Provox NID had the most residue on the distal end of the prosthesis with the least residue on the Provox Vega. Post hoc analysis of voice prosthesis residue on thin liquids is shown in Table 7. Post hoc analysis of voice prosthesis residue on soft indicated no significant mean differences. This post hoc test therefore contradicts the main (omnibus) test. This can occur when there are two groups of voice prostheses that are different when considered in the omnibus test (Type III tests of fixed effects) but are only nearly different to the others in the post hoc test.

**Table 7:** Post hoc analysis—voice prosthesis residue on thin liquids

Pairs	P (*p* < 0.5)	Mean difference	Prosthesis with higher score
Blom Singer LP versus Blom Singer Classic ID	0.049	9.33	Blom Singer Low Pressure
Blom Singer Classic ID versus Blom Singer Advantage	0.0001	13.76	Blom Singer Advantage
Blom Singer Classic ID versus Provox NID	0.015	8.19	Provox NID
Blom Singer Classic ID versus Provox Vega	0.024	10.48	Provox Vega

Type III tests of fixed effects also indicated a significant difference between voice prostheses in terms of post swallow residue in the upper esophagus on soft only (*p* ≤ 0.001). On soft, the Blom Singer duckbill had the most residue in the upper esophagus, with the least residue on the Blom Singer low pressure.

Post hoc analysis for residue in the upper esophagus on soft is indicated in (Table 8).

**Table 8:** Post hoc analysis—esophageal residue on soft

Pairs	P (*p* < 0.5)	Mean difference	Prosthesis with higher score
Blom Duckbill versus Provox Vega	0.001	19.31	Blom Singer Duckbill
Provox NID versus Provox Vega	0.041	13.42	Provox NID

## Discussion

This study aimed to explore whether voice prostheses have an effect on swallowing function in terms of patient self-evaluation, and expert rater consensus of residue on the prostheses and in the upper esophagus. The findings of this study indicated no significant difference in ratings between prostheses for participants. The Blom Singer Low Pressure and Blom Singer Classic Indwelling were most frequently chosen by expert raters as preferable for swallowing when a consensus was reached, but for most participants no expert consensus was reached. The least amount of residue was found on the Blom Singer Classic Indwelling voice prosthesis with thin liquids, and on the Provox Vega with soft consistency. The least amount of residue in the upper esophagus was found on the Blom Singer Low Pressure with soft consistency only.

A limited number of studies [2, 9, 22, 23] have examined patient perception of voice prosthesis function but none have investigated this in relation to swallowing. Although some individual participants commented strongly that certain voice prostheses were easier to swallow with than others, the analysis of the group as a whole indicated that scores were equally distributed and no one voice prosthesis or prostheses emerged as preferential for swallowing. A study [2] of patient perception of indwelling voice prostheses for the purposes of voice noted a high degree of diversity in patient responses and indicated that patients do not perceive all indwelling prostheses as equal. The choice of voice prosthesis for a patient is usually made based only expert clinical opinion rather than patient preference. As patients themselves sometimes report significant differences amongst voice prostheses for both voice and swallow, it may be helpful to incorporate patient choice when placing a new voice prosthesis. This study was limited by the fact that voice prostheses were not in place for a prolonged length of time. It is possible that research focused on patient self-evaluation of swallow performance with a voice prosthesis over a number of days or weeks while eating meals at home, or in social situations, may have yielded a different result. This potential topic of future research may also help illuminate whether altered swallow function as a result of voice prosthesis change is an important issue for laryngectomy patients.

Similarly the expert raters did not reach a consensus on the best prosthesis for swallow for most participants. The Blom Singer Low Pressure and Blom Singer Classic Indwelling were most frequently chosen as best prosthesis for swallow suggesting that these two prosthesis designs may offer some advantages for some patients. Nevertheless, these results should be interpreted cautiously given the subjective nature of the task. This is the first study that has examined expert rater analysis of patient swallow performance with different voice prostheses; further research may illuminate this area in order to improve patient outcomes post laryngectomy.

The final objective of this study was to investigate whether there is a difference between voice prostheses in terms of the degree of residue on the voice prosthesis and in the upper esophagus as judged by expert raters. It is worth considering that factors other than the voice prosthesis, such as anatomy and type and amount of food consistency, can influence the amount of residue experienced by a laryngectomy patient. In this study, the effect of voice prostheses was measured across the same group of participants with both the type and amount of food consistencies strictly controlled. Results indicated a significant difference in amount of residue with thin liquids on the voice prosthesis and with soft on both the voice prosthesis and in the esophagus.

Both the Provox Vega and the Blom Singer Classic Indwelling were found to have least residue. In common with the Provox NID and the Blom Singer Low Pressure voice prostheses, the esophageal end of the Provox Vega has a flap surrounded by a hood that protrudes slightly into the esophagus (see Fig. 2). However, the Provox Vega contains a recessed and angled flap, which is totally encased by the prosthesis hood and is designed to minimise direct exposure of the prosthesis to the esophagus. It is possible that this characteristic may help reduce residue. The Blom Singer Classic Indwelling features an entirely flat esophageal flange. This feature is shared with the Blom Singer Advantage voice prosthesis. However, the Blom Singer Advantage has a larger and textured esophageal flange, which may influence residue accumulation. The most residue was found in the esophagus on the Blom Singer duckbill. This prosthesis has a “bullet”-shaped nose containing a slit valve rather than a flap valve. Depending on the amount of space between the anterior and posterior walls of the esophagus, this prosthesis sometimes appeared to touch the posterior wall of the esophagus obstructing bolus flow during swallowing. It is possible that the characteristic of a small flat esophageal flange, which does not protrude into the esophagus, may help to minimise residue because there are no areas of the flange, such as a hood, which can catch residue. It appears likely that individual characteristics of different voice prostheses such as configuration and size of esophageal flange may influence swallowing behaviour.

## Limitations

Limitations of this study include the lack of good inter-rater reliability for all questions on the rating scale used by experts to judge swallow on each voice prosthesis. Previous studies [31–33] have identified poor inter-rater reliability when judging instrumental swallowing evaluations and have highlighted the largely subjective nature of the task. Further research is required to improve reliability of rating scales used to judge swallowing across different instrumental evaluation tools and patient etiologies. The use of FEES as a dysphagia evaluation tool was advantageous to this study because of the lack of radiation exposure and the number of swallow evaluations required for each participant. However, it is possible that the use of an alternative dysphagia evaluation tool may have yielded a different result. A further limitation of this study is that voice prostheses remained in situ for a relatively short period of time. Future work to examine the longer-term effects of voice prostheses on swallow may prove beneficial.

## Conclusions

Neither laryngectomy participants nor expert raters consistently identified a single ‘best’ prosthesis for swallowing. However, individual participants did notice differences and so individual choice when trialling prostheses may be important. As there is little agreement among expert raters, it would appear that patients’ subjective experience of swallowing is the most appropriate criteria to use in making the choice of prosthesis.

This study provides some preliminary evidence that the Blom Singer Classic Indwelling and Provox Vega voice prostheses may be associated with lower residue levels than other voice prostheses as measured by expert raters. Both these prostheses have characteristics that may help minimise residue. However, further research is required to investigate the characteristics, if any, of individual voice prostheses and how these characteristics might affect swallowing behaviour.
